# Effect of season and diet on heart rate and blood pressure in female red deer (*Cervus elaphus*) anaesthetised with medetomidine-tiletamine-zolazepam

**DOI:** 10.1371/journal.pone.0268811

**Published:** 2022-06-07

**Authors:** Hanna Rauch, Friederike Pohlin, Joy Einwaller, Manuela Habe, Kristina Gasch, Anna Haw, Walter Arnold, Gabrielle Stalder, Johanna Painer

**Affiliations:** Department of Interdisciplinary Life Sciences, Research Institute of Wildlife Ecology, University of Veterinary Medicine, Vienna, Austria; Universitat Autònoma de Barcelona, SPAIN

## Abstract

Temperate zone ungulates like red deer (*Cervus elaphus*) show pronounced seasonal acclimatisation. Hypometabolism during winter is associated with cardiovascular changes, including a reduction in heart rate (*f*_H_) and temporal peripheral vasoconstriction. How anaesthesia with vasoactive substances such as medetomidine affect the seasonally acclimatised cardiovascular system is not yet known. We anaesthetised eleven healthy female red deer with medetomidine (0.1 mg/kg) and tiletamine/zolazepam (3 mg/kg) twice in winter (*ad libitum* and restricted feed) and in summer (*ad libitum* and restricted feed), with a two-week washout-period in-between, to test for the effect of season, food availability and supplementation with omega-3 or omega-6 polyunsaturated fatty acid (PUFA) on *f*_H_ and arterial blood pressure (ABP) during anaesthesia. Six animals received pellets enriched with omega-6 fatty acids (FA), and five animals with omega-3 FA. Anaesthesia significantly decreased *f*_H_ in summer but not in winter and ABP was lower in winter (*p* < 0.05). The combination of omega-6 FA enriched pellets and food restriction resulted in a lower *f*_H_ and higher ABP during anaesthesia with more pronounced changes in winter (*p* < 0.001). Our results demonstrate that season, food availability and type of PUFA supplementation in red deer affect the cardiovascular system during anaesthesia.

## Introduction

Many temperate zone mammals employ physiological acclimatisation during winter to cope with low temperatures and poor food availability [1–4]. Hibernation and daily torpor are two such well-known physiological acclimatisation mechanisms, characterised by extended periods of metabolic depression during winter and high metabolic activity during summer, especially in small mammals [5]. Larger, non-hibernating mammals, such as red deer, also show a degree of metabolic reduction during winter [3] as evidenced by a lower heart rate (*f*_H_) [4]. Together with this lower *f*_H_, red deer also have enhanced peripheral vasoconstriction leading to cooler peripheral body parts in winter [4,6]. In hibernators, a decrease in arterial blood pressure (ABP) occurs concurrently with a decreased metabolic rate [7,8]. Whether non-hibernating hypometabolic mammals also show a reduction in ABP during winter is not yet known.

In addition to these primary physiological adjustments, coordinated predominantly by endogenous signals entrained to the photoperiod, changes in environmental conditions, such as food availability and quality, are important secondary triggers that affect seasonal acclimatisation [6,9,10]. Red deer voluntarily reduce their food intake (VFI) during the winter as a result of reduced energy expenditure, organ size and metabolic rate [11,12]. The reduction of VFI in winter persist under experimental *ad libitum* feeding, indicating endogenous regulation [11]. However, when food is restricted beyond the VFI during winter, the endogenous down-regulation of *f*_H_ is further enhanced [11].

On a molecular level, the type of dietary polyunsaturated fatty acids (PUFA) is thought to play a role in seasonal acclimatisation [13,14]. The incorporation of fatty acids (FA) with lower melting points likely contributes to maintaining membrane fluidity and integrity during low temperatures [13,15,16]. Hibernators that received a diet rich in omega-6 FA before the hibernation period, exhibited longer torpor bouts, lower minimum body temperatures and less energy expenditure during winter [17,18]. Similarly, it is assumed that increased incorporation of omega-6 FA into phospholipids in peripheral skeletal muscle during winter might allow for peripheral hypothermia in red deer [4].

Temperate zone mammals, such as red deer, often require anaesthesia or chemical immobilisation for management, medical interventions or research purposes. Immobilisation, most commonly induced with a combination of an α2-adrenoceptor agonist, such as medetomidine, and a dissociative anaesthetic combination such as tiletamine-zolazepam [19] also results in severe cardiovascular changes including hypertension or bradycardia [20].

How the seasonal- and dietary-induced cardiovascular changes influence these more acute drug-induced physiological changes during immobilisation is not yet known.

Therefore, the aim of this study was to investigate the effect of two different seasons (summer vs. winter) and dietary treatments (food restriction and type of PUFA supplementation) on *f*_H_ and ABP in red deer hinds anaesthetised with medetomidine-tiletamine-zolazepam. We hypothesised that *f*_H_ and ABP would be higher during summer than during winter and lower in deer on a restricted, or omega-6 FA rich diet.

## Material and methods

### Ethical statement

Captive adult female red deer were regularly anaesthetised in the course of the FWF-research project P 30061 “Polyunsaturated fatty acids and seasonal acclimatisation.” The study was approved by the Ethics and Animal Welfare Committee of the University of Veterinary Medicine, Vienna in accordance with the University’s guidelines for Good Scientific Practice and authorised by the Austrian Federal Ministry of Education, Science and Research (permit number: 68.205/0021-WF/V/3b/2017) in accordance with the current legislation. Anaesthesia monitoring data of eleven animals were retrospectively analysed for this study.

### Study area and animals

The animals were living, as part of a general deer population consisting of 37 females and one adult stag, under close to natural conditions in a 45 ha enclosure located at the Research Institute for Wildlife Ecology of the University of Veterinary Medicine, Vienna (48.22°N, 16.28°E). All deer, included in the study, were reproducing at the time of the experiment. The area contained a meadow (3 ha), which was surrounded by mixed beech and oak forest. In addition to pellets, provided by an automatic feeding station [11], the animals foraged on natural vegetation. The animals were trained by positive reinforcement to tolerate close contact to humans to provide stress-free handling and anaesthesia induction. Their age ranged from 7–11 (mean 8.2 ± 1.0 standard deviation) years and their weight, measured by a scale located underneath the feeding station the day before each anaesthesia, was 155 ± 24 (range 133–217) kg during summer and 141 ± 18 (range 110-160) kg during winter. Air temperature was recorded every 10 min throughout the study period in a weather station located in the study area. Rumen temperature (T_R_) was measured every 3 min via a rumen logger as described earlier (e.g. [11,21]).

### Baseline heart rate measurement

Baseline *f*_H_ were continuously measured by a rumen transmitter [21]. This transmitter recorded *f*_H_ at rest every three minutes and transmitted this information via a short-range telemetry (100 kHz) to a receiver and storage unit located in a collar on the animal’s neck. These measurements were averaged daily within individuals over a time window of one week before immobilisations took place.

### Dietary treatments

**Fig 1:**
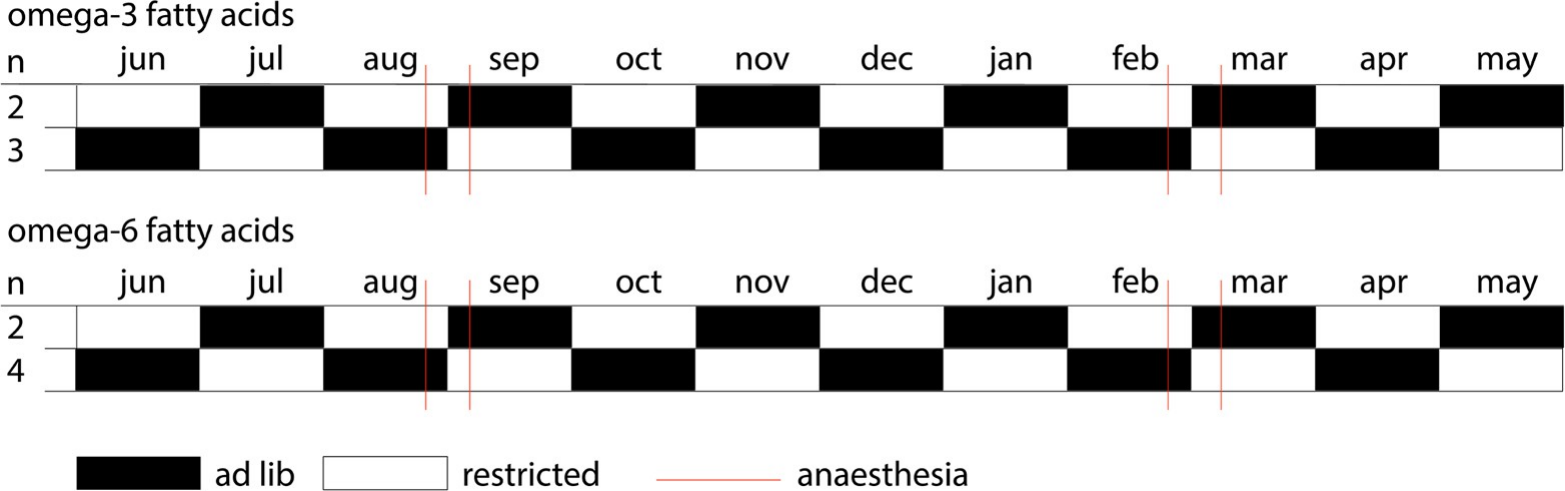
Experimental feeding schedule. Animals received either food pellets supplemented with omega-3 α-linolenic acid (five animals), or omega-6 linoleic acid (six animals), by immersing the pellets in 5% w/w linseed oil or sunflower seed oil, respectively. Feeding regimes alternated monthly between *ad libitum* (black bar) and restricted (white bar) feeding. During each season animals were anaesthetised twice (red line). Numbers in the first column (n) represent the number of animals assigned to a feeding schedule.

Deer were fed a daily individual amount of one of two pellet types (Fig 1). A transponder within the animal’s ear tag and a scale located underneath the feeding station (Schauer, Prambachkirchen, Austria) allowed for identification and body weight assessment of each individual. Animals were fed commercially available red deer pellets either immersed in 5% w/w linseed oil, rich in omega-3 α-linolenic acid (five animals), or in 5% w/w sunflower seed oil, rich in omega-6 linoleic acid (six animals), respectively. The pellet type, an individual was assigned to, remained consistent throughout the study period (Fig 1). However, changes in the feeding regime were made. In all individuals, unlimited access to pellets (*ad libitum*) was alternated with food restriction every month. During food restriction, each animal received 20% of the average amount of pellets consumed per day during the *ad libitum* period (Fig 1).

### Anaesthesia

Each animal underwent two anaesthesia events in August 2018 and two in February 2019, with a two-week washout period between subsequent treatments. Between subsequent anaesthetic events, feeding regimes changes from *ad libitum* to restricted feeding, or vice versa (Fig 1).

For immobilisation, the deer were called into a crate. Anaesthesia was induced with medetomidine (0.1 mg/kg; Medetomidine-HCL 2%, magistral formulation, Richter Pharma AG, Vienna, Austria) and tiletamine-zolazepam (3 mg/kg; Zoletil 100, Virbac, Glattbrugg, Switzerland) administered intramuscularly (IM) via hand injection.

Once immobilisation was achieved, deer were positioned in lateral recumbency, their eyes lubricated (Oleovit Augensalbe, Laevosan, Linz, Austria) and covered by a blindfold. Oxygen was administered via nasal insufflation (3 L/min) and a 16-gauge catheter (Vasofix Safety, Ø 1,7 x L 50 mm, B. Braun Austria GesmbH, Maria Enzersdorf, Austria) was inserted into a jugular vein to provide a constant rate of crystalloid infusion (6 mL/kg/hr; NaCl 0,9%, B. Braun Melsungen AG, Melsungen, Germany) throughout the procedure. To mitigate hypo- or hyperthermia during anaesthesia, red deer were covered with an insulating sleeping bag during the winter, or positioned in the shade during the summer.

A multiparameter anaesthetic monitor (Root™ Radical-7; rainbow RC-12; Phasein ISA™; Nomoline™; M-LNCS™; Masimo Corporation, Irvine, CA, USA) was used to continuously measure peripheral oxygen haemoglobin saturation (SpO_2_), respiratory rate and end-tidal carbon dioxide (PE’CO_2_) concentration. For the latter, a shortened 6-mm internal diameter cuffed endotracheal tube (Covetrus AT GmbH, Brunn am Gebirge, Austria) was placed into the ventral meatus of the animal’s nostril.

To allow for the continuous measurement of *f*_H_ and ABP during anaesthesia, a 22-gauge catheter (Vasofix Safety, Ø 0,9 x L 45 mm, B. Braun Austria GesmbH, Maria Enzersdorf, Austria) was aseptically placed into an auricular artery. Hear rate and direct systolic (SAP), mean (MAP) and diastolic (DAP) arterial blood pressures were measured using a patient-side pressure monitor (IntraTorr^™^; IntraVital, UK) that was connected with noncompliant tubing to an electronic strain-gauge transducer zeroed to atmospheric air pressure at the level of the right atrium. Digital recording of these measurements started from 25 minutes after induction and was performed for 30 minutes in intervals of 15 seconds.

Anaesthesia was terminated 60 minutes after induction by antagonising medetomidine with atipamezole (0.5 mg/kg; Narco Stop, Richter Pharma AG, Wels, Austria) administered IM. Each animal was kept under observation until fully recovered and observed over 24 hours for any signs of re-narcotisation.

### Statistical analyses

For *f*_H_ and ABP, values outside the described physiological range for red deer (30–100 beats/minute for *f*_H_ and 60–240 mmHg for ABP) [20] were removed from the database by visual inspection. Furthermore, incomplete data sets were excluded from the analysis.

Linear mixed modelling for repeated measures (nlme package [22]) was performed using the software package R for Mac OS X [23]. Anaesthetic time (seconds), season (summer or winter), food regime (restricted or *ad libitum*), pellet type (omega-6 or omega-3 FA), body mass (kg), age (year), and interactions between season, food regime, pellet type and time were used as independent variables. Additionally, we included an autoregressive structure of order 1 (corAR1 function; [24]) in all models to account for intraindividual temporal correlations.

To account for repeated observations within individuals, we included a random intercept per individual when testing the effect of these independent variables on *f*_H_ and ABP (dependent variables). To find the best subset of fixed effect, a model selection was performed by calculating Akaike`s information criterion (AICc) values using the dredge function implemented in the MuMIn package [25]. The final models were fitted with restricted maximum likelihood. To assess the amount of variation explained by each model, we used the function r.squared GLMM (from MuMIn package [25]) to calculate the coefficients of determinations (R^2^) for fixed effects (marginal R^2^) and fixed and random effects combined (conditional R^2^). Plots and a histogram of the model residuals were performed to check for homoscedasticity and normal distribution. To visualise the effect of the independent variables over time and between groups, we fitted another model in which we included time as factor. We computed the predicted marginal means of each group at every time point using the lsmean package and finally plotted these results (ggplot2 package [26]).

Changes in cardiovascular parameters over time between treatments (e.g. summer vs. winter) were analysed by using a post-hoc test (Tukey HSD, lsmeans package [27]). Additionally, the *f*_H_ of anaesthetised red deer, was compared to baseline *f*_H_ within treatments, where a *p*-value < 0.05 was considered as statistically significant. Means are reported with standard error of the mean (SEM).

## Results

Overall, twenty-five anaesthetic events of eleven animals were included. In eight of these anaesthetic events, the start of direct ABP and *f*_H_ monitoring was delayed by 1 to 11 minutes due to logistical reasons in setting up the sampling line. Record of cardiovascular parameters, logged at intervals of 15 seconds, revealed up to 120 consecutive values for *f*_H_ and ABP for each anaesthetic event.

Season and dietary treatment contributed significantly to variation in cardiovascular parameters. Results are presented in Table 1 for *f*_H_, Table 2 for SAP and S1 and S2 Tables for MAP and DAP, respectively. Models with the lowest AIC had an explanatory power of 89% (fixed effects 46%), 88% (fixed effect 44%), 80% (fixed effect 35%) and 79% (fixed effect 37%) for SAP, MAP, DAP and *f*_H,_ respectively. Results of the post hoc test are presented as mean and SEM.

**Table 1:** Factors explaining the variation in heart rate of anaesthetised female red deer. Results of the best fitted linear mixed effect model explaining *f*_H_ of female red deer (*Cervus elaphus*, n = 11) during anaesthesia with 0.1 mg/kg medetomidine and 3 mg/kg tiletamine-zolazepam. Animals were anaesthetised twice in winter (*ad libitum* and restricted feed) and twice in summer (*ad libitum* and restricted feed) and received pellets enriched with omega-3 FA (n = 5) or omega-6 FA (n = 6) (type of PUFA supplementation).

Random effect	Std. Error			
Subject	6.13			
Predictor	Coefficient	Std. Error	Confidence Interval	*p*- value
(Intercept)	37.83	6.36	25.35 to 50.30	< 0.001
Food regime[restricted]	-8.5	0.87	-10.21 to -6.79	< 0.001
PUFA type [omega-6 FA]	-7.54	3.97	-16.69 to 1.61	0.094
Season[Winter]	3.53	1.07	1.43 to 5.63	0.001
Time	0.02	0.01	0.00 to 0.03	0.021
Age	1.7	0.81	-0.17 to 3.56	0.069
Body mass	-0.05	0.01	-0.07 to -0.03	< 0.001
Food regime [restricted]*PUFA type [omega-6 FA]	7.14	0.90	5.37 to 8.9	< 0.001
Season[Winter]*Food regime [restricted]	-2.85	0.97	-4.75 to -0.94	0.003
Season[Winter]*PUFA type [omega-6 FA]	-2.59	0.92	-4.39 to -0.79	0.005

conditional R^2^ = 79%, marginal R^2^ = 37%.

**Table 2:** Factors explaining the variation in direct systolic arterial pressure of anaesthetised female red deer. Results of the best fitted linear mixed effect model explaining systolic arterial blood pressure of female red deer (*Cervus elaphus*, n = 11) during anaesthesia with 0.1 mg/kg medetomidine and 3 mg/kg tiletamine-zolazepam. Animals were anaesthetised twice in winter (*ad libitum* and restricted feed) and twice in summer (*ad libitum* and restricted feed) and received pellets enriched with omega-3 FA (n = 5) or omega-6 FA (n = 6) (type of PUFA supplementation).

Random effect	Std. Error			
Subject	11.81			
Predictor	Coefficient	Std.Error	Confidence Interval	*p*-value
(Intercept)	100.43	13.28	74.39 to 126.46	< 0.001
Food regime[restricted]	-0.82	1.40	-3.57 to 1.93	0.558
PUFA type [omega-6 FA]	-15.53	7.70	-33.29 to 2.23	0.079
Season[Winter]	-8.02	1.82	-11.59 to -4.45	< 0.001
Time	-0.09	0.01	-0.1 to -0.08	< 0.001
Age	-0.21	1.59	-3.88 to 3.47	0.900
Body mass	0.43	0.043	0.34 to 0.51	< 0.001
Food regime[restricted]*PUFA type [omega-6 FA]	14.05	1.49	11.12 to 16.97	< 0.001
Season[Winter]*Food regime[restricted]	-4.18	1.48	-7.09 to -1.28	0.005
Season[Winter]*PUFA type [omega-6 FA]	0.89	1.73	-2.5 to 4.28	0.608

conditional R^2^ = 89%, marginal R^2^ = 46%.

### Effect of season

**Fig 2:**
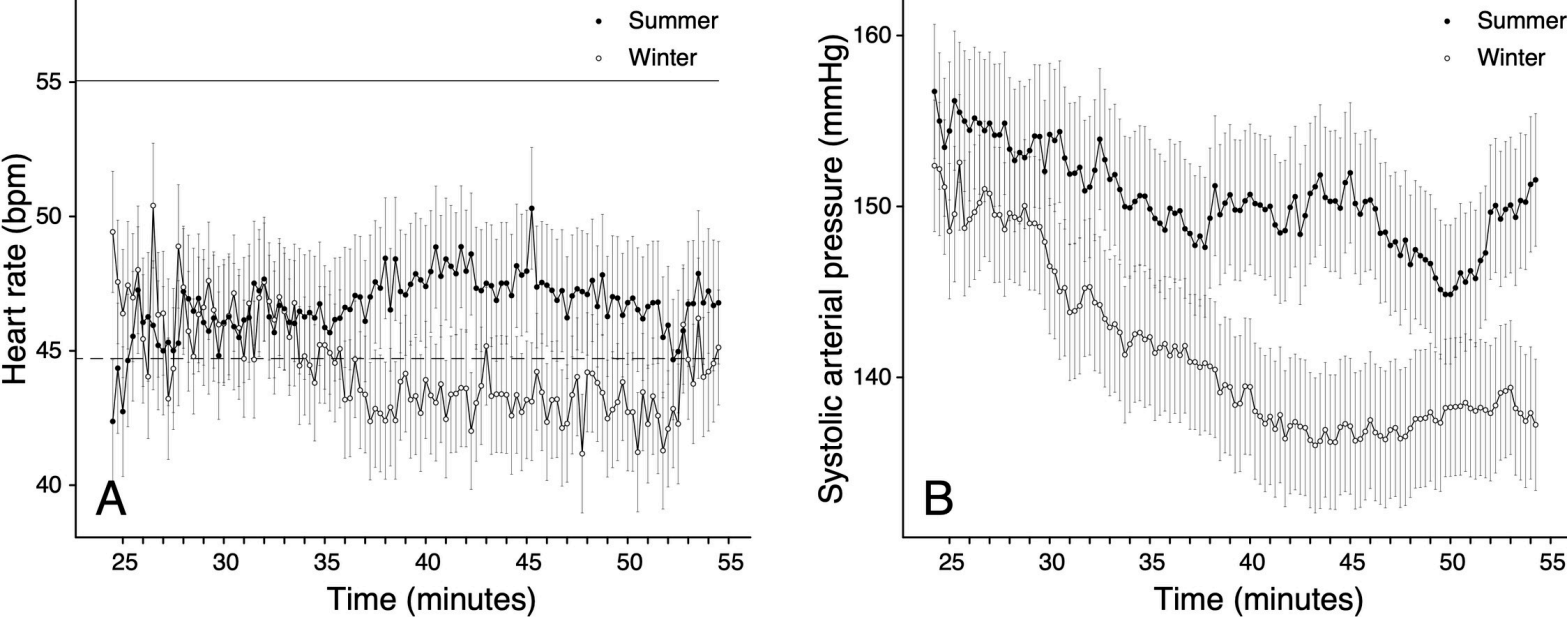
Seasonal differences of heart rate (A) and direct systolic arterial pressure (B) of female red deer (*Cervus elaphus)*. Deer (n = 11) were anaesthetised with 0.1 mg/kg medetomidine and 3 mg/kg tiletamine-zolazepam. Heart rate and SAP were measured from minute 25 to 55 after anaesthesia induction (means ± standard error of the mean). Baseline heart rate values for summer (solid) or winter (dashed) season are represented by a horizontal line.

Cardiac activity varied throughout the seasons with a baseline *f*_H_ of mean 55 ± 2 (SEM) beats/minute during the summer and 45 ± 2 beats/minute during the winter (Fig 2A). In the summer, *f*_H_ decreased significantly after inducing anaesthesia (*p* < 0.001) and, despite a mild increase over time, remained below baseline values until the end of anaesthesia (Fig 2A). In winter, the divergence of *f*_H_ from baseline values was not as pronounced and a decreasing trend was noted over time. Overall, *f*_H_ in anaesthetised deer was higher in summer compared to winter (Fig 2A).

Season strongly affected the ABP in anaesthetised red deer (Tables 2, S1 and S2) (*p* < 0.05). ABP was higher in summer compared to winter with approximately 150 ± 4, 108 ± 3 and 128 ± 3 mmHg for SAP (Fig 2B), DAP (S1A Fig) and MAP (S1B Fig) in the summer and 141 ± 4, 104 ± 10 and 121 ± 3 mmHg in winter (Figs 2B & S1). Time had a strong and significant effect on ABP (*p* < 0.001), which gradually decreased during anaesthesia in both seasons. Mean daily body temperature of the animals was 38.8 ± 0.04°C in winter and 39.0 ± 0.04°C in summer and did not differ significantly between the seasons, or between the two anaesthesia timepoints in each season respectively. Mean daily air temperature differed significantly between the seasons, and was 2.95 ± 0.11°C in winter and 24.7 ± 0.12°C in summer.

### Effect of dietary treatment

#### Food regime

**Fig 3:**
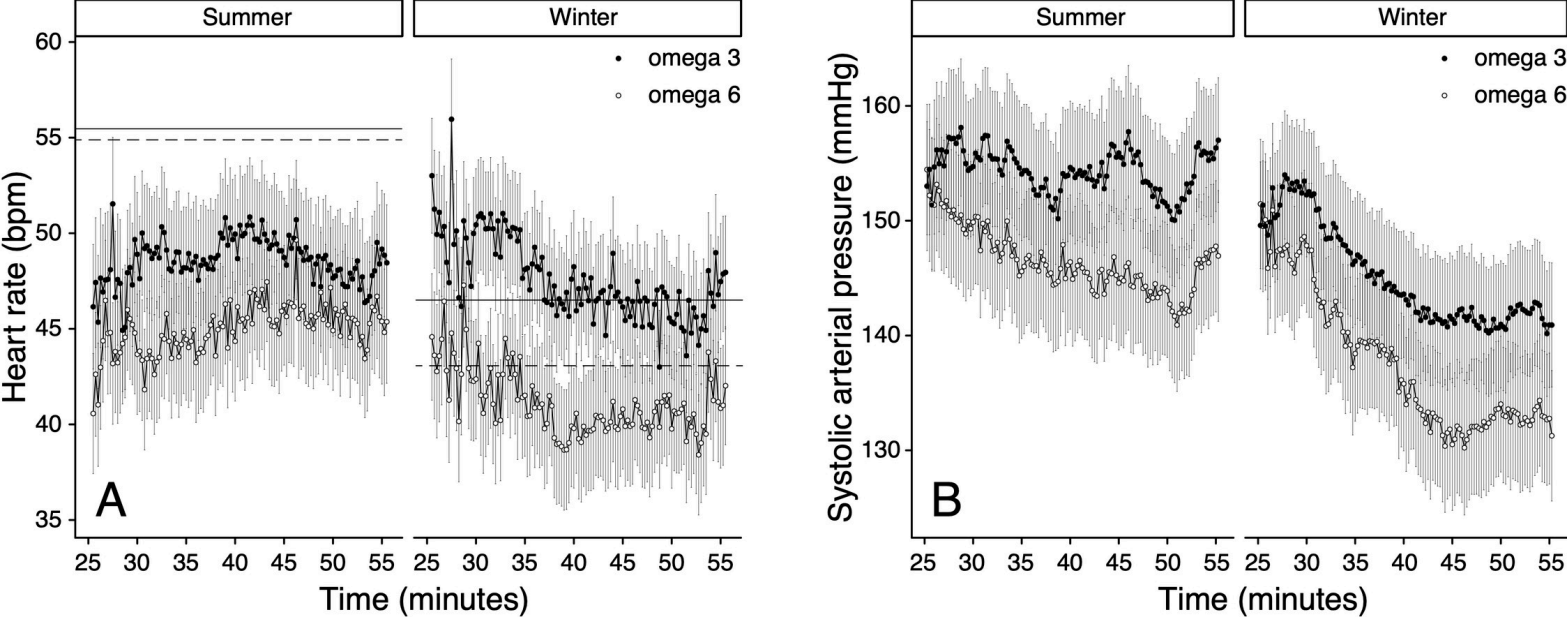
Seasonal differences during periods of unrestricted (*ad libitum*) and restricted food intake on heart rate (A) and direct systolic arterial pressure (B) in female red deer (*Cervus elaphus*, n = 11). Heart rate and SAP were measured from minute 25 to 55 after anaesthesia induction (means ± standard error of the mean). Baseline heart rate values for periods of unrestricted (*ad libitum*) (solid) or restricted (dashed) pellet intake are represented by a horizontal line. (See legend Fig 2. for medetomidine-tiletamine-zolazepam doses).

Deer fed with a restricted diet had a lower *f*_H_ and higher ABP during anaesthesia in the given season, than the ones who had *ad libitum* access to feed (Figs 3A, 3B and S2). The interaction between food regime and season was significant for *f*_H_ (*p* = 0.003), SAP (*p* = 0.005), MAP *(p* = 0.007*)* and DAP (*p* = 0.003) (Tables 1, 2, S1 and S2).

#### Type of PUFA supplementation

**Fig 4:**
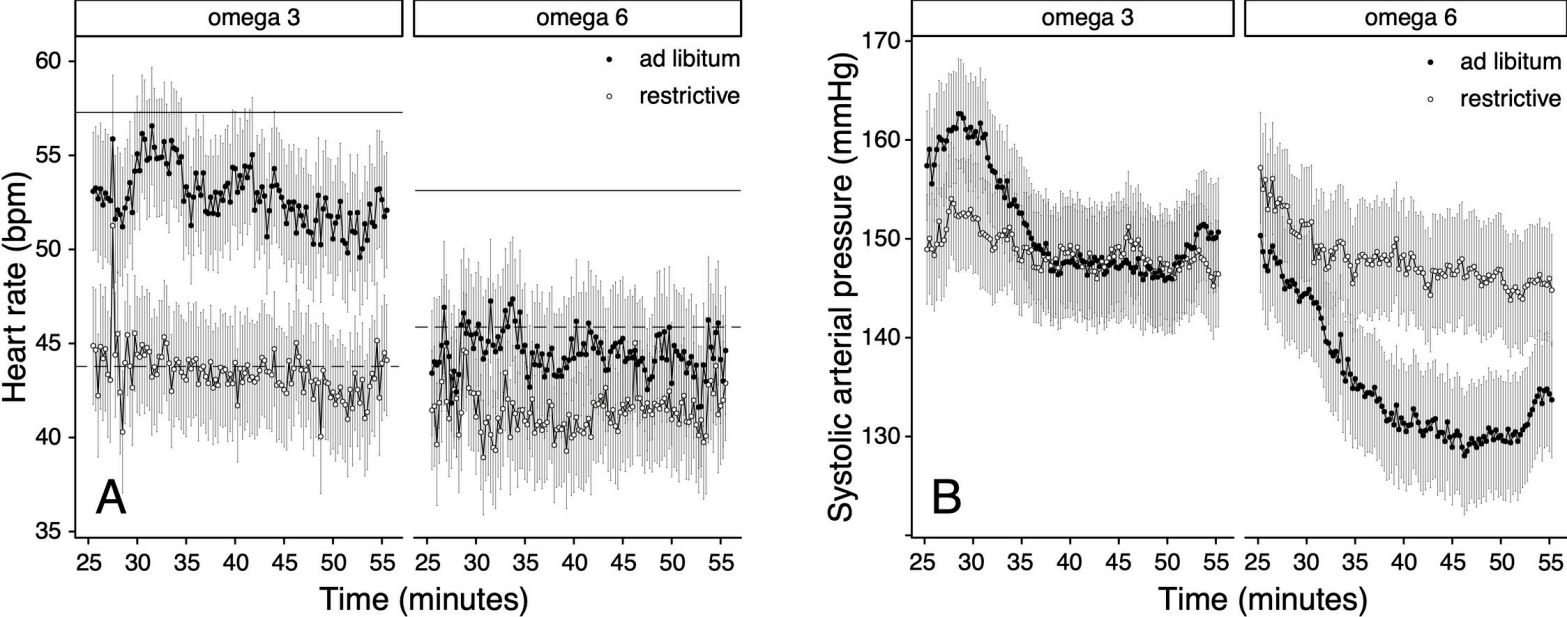
The influence of food regime and type of PUFA supplementation (omega-6 FA (n = 6) or omega-3 FA (n = 5) rich pellets) on heart rate (A) and direct systolic arterial pressure (B) in female red deer (*Cervus elaphus*, n = 11). Heart rate and SAP were measured from minute 25 to 55 after anaesthesia induction (means ± standard error of the mean). Baseline heart rate values for periods of unrestricted (*ad libitum*) (solid) or restricted (dashed) pellet intake are represented by a horizontal line. (See legend Fig 2. for medetomidine-tiletamine-zolazepam doses).

**Fig 5:**
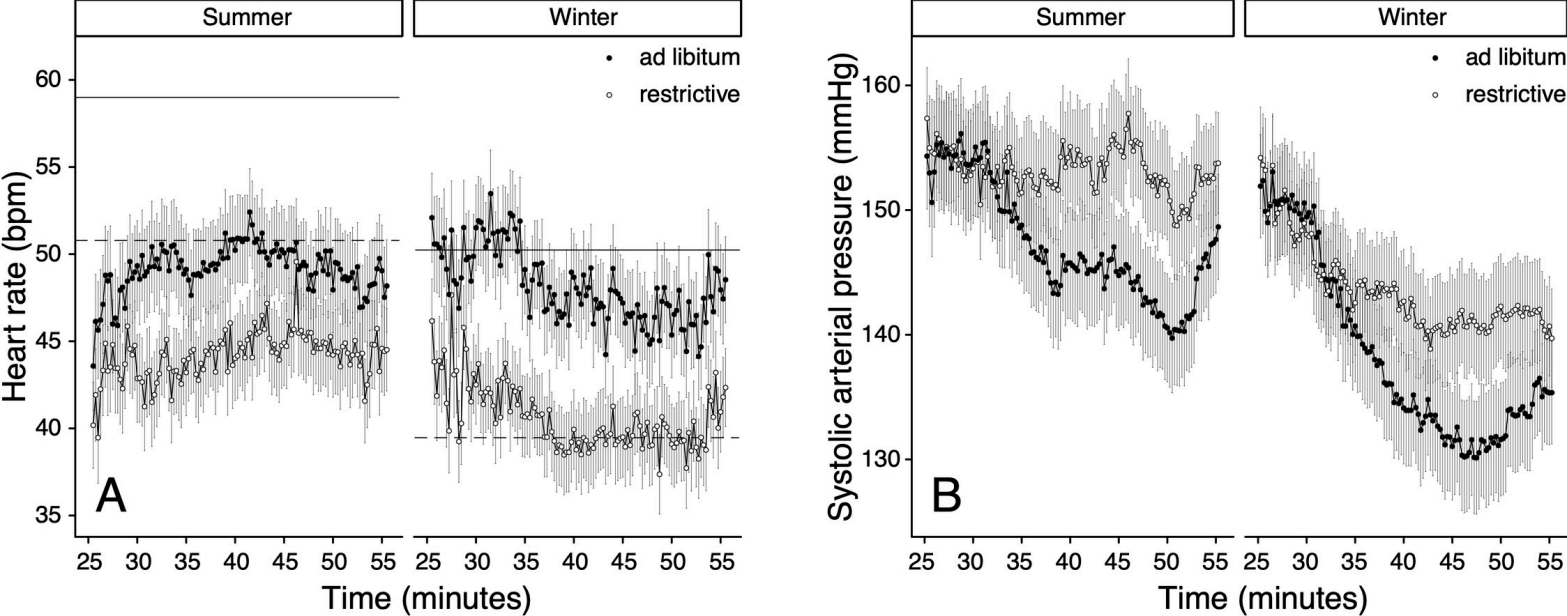
The influence of alternating type of PUFA supplementation (omega-6 FA (n = 6) or omega-3 FA (n = 5) rich pellets) on heart rate (A) and direct systolic arterial pressure (B) in two different seasons (summer vs. winter) in female red deer (*Cervus elaphus*, n = 11). Heart rate and SAP were measured from minute 25 to 55 after anaesthesia induction (means ± standard error of the mean). Baseline heart rate values for omega-3 (solid) or omega-6 (dashed) enriched pellet intake are represented by a horizontal line.

Type of PUFA supplementation influenced *f*_H_ and ABP differently during periods of food restriction and periods of *ad libitum* feeding. The combination of omega-6 FA enriched pellets and food restriction resulted in a lower *f*_H_ (Fig 4A) and higher ABP (Figs 4B and S3) during anaesthesia. During both seasons, deer fed with omega-6 FA enriched pellets had a lower *f*_H_ (Fig 5A) and ABP (Figs 5B and S4) compared to deer fed with omega-3 FA enriched pellets. There was a significant interaction of type of PUFA supplementation with food regime for *f*_H_ (*p* < 0.001), SAP (*p* < 0.001) MAP (*p* < 0.001), and DAP (*p* < 0.001) and type of PUFA supplementation with season for *f*_H_ (*p* = 0.005), MAP (*p* = 0.002), and DAP (*p* = 0.005) (Tables 1, 2, S1 and S2).

## Discussion

This study compared *f*_H_ and ABP in female red deer anaesthetised with medetomidine-tiletamine-zolazepam across two seasons and different feeding regimes and type of PUFA supplementation. Red deer experienced lower *f*_H_ and ABP during the winter compared to the summer. Deer on a restricted diet and/or receiving omega-6 FA rich pellets had a lower *f*_H_ and higher ABP than deer fed *ad libitum* and/or receiving omega-3 FA rich pellets. These results indicate that seasonal and dietary cardiovascular changes are present during anaesthesia and should be considered when working with seasonal species.

Seasonal differences in *f*_H_, with lower values during winter, are well-known cardiovascular adaptations in hibernating and non-hibernating mammals [6,28–30]. Similar to our study, Arnold et al., 2004 [4] reported a 60% decrease in *f*_H_ in unsedated red deer during winter compared to summer. A concurrent decrease in blood pressure has been recorded in only a few other species that also undergo seasonal adaptations including ground squirrels [31], Syrian hamsters [8], marmots [32] and laboratory mice [33]. Our finding that overall *f*_H_ and ABP are reduced in red deer hinds during winter, compared to summer, is in line with these previous studies and likely reflects a decrease in metabolic rate and energy expenditure [8,34].

However, our animals were anaesthetised when these measurements were taken and, although it appears that seasonal changes persisted during anaesthesia, the anaesthetic drugs also influenced cardiovascular changes. Hypertension and reflex-bradycardia are well-known side effects of medetomidine administration [35,36] and tiletamine might further worsen the hypertension [37]. As expected, IM administration of 0.1 mg/kg medetomidine and 3 mg/kg tiletamine-zolazepam markedly increased ABP in all animals. During both seasons, direct SAP, MAP, DAP were considerably elevated compared to reference intervals reported for unsedated ungulates [38].

Interestingly, the reflex-bradycardia, defined as a 20% reduction below resting *f*_H_ [39], appeared to occur only in summer. The baroreflex is a vagally mediated cardiac reflex that occurs in response to an elevated ABP and causes the *f*_H_ (“reflex bradycardia”) and thus, the ABP, to decrease [40]. By placing an arterial catheter in the abdominal aorta, Horwitz et al. (2013) [8] continuously recorded ABP in Syrian hamsters during hibernation and calculated the baroreflex sensitivity in this species. The study suggested that the baroreflex-control of the *f*_H_ regulates ABP during hibernation, but with a decreased set-point during winter [8]. Similarly, a decreased baroreflex sensitivity together with a generally lower ABP during winter, could explain the missing reflex-bradycardia after medetomidine administration in our red deer. Further studies in awake animals need to be performed in order to confirm these assumptions.

Deer included in our study were all reproductively active and pregnant throughout the data collection in February 2019 which may have further influenced cardiovascular variables. During pregnancy, cardiovascular changes, defined by elevated heart rate values and an increase in stroke volume, may occur. Together with a decrease in peripheral vascular resistance, produced by plasma oestrogens, this will lead to an increase in cardiac output while ABP remains unchanged [41]. Determining the effect of pregnancy was out of the scope of our study but would be of interest in future research.

Metabolic rate is highly correlated with energy expenditure and food consumption [10]. In our study, restricted food intake contributed to a lower *f*_H_ in anaesthetised red deer hinds. These results concur with recent studies, where experimental food restrictions caused a decrease in *f*_H_ over a period of eight days in red deer during the winter [11]. We also found that ABP was higher in anaesthetised red deer hinds on a restricted diet compared to those that received food *ad libitum*. These changes in ABP were likely caused by changes in peripheral vascular resistance associated with thermoregulation [42]. In response to increased or decreased body temperature, peripheral blood flow is modified through sympathetic-mediated vasodilation or vasoconstriction [43]. During restricted food intake, a decrease in endogenous heat production may cause a decrease in body temperature [11] and thus, increased peripheral vasoconstriction [4] and ABP. We believe that, in our deer, this effect was even more pronounced during the winter, because of the lower environmental temperatures.

Lastly, type of PUFA supplementation influenced *f*_H_ and ABP in our animals. In food restricted deer and during winter, pellets enriched with omega-6 FA led to lower *f*_H_ and higher ABP during anaesthesia, compared to animals receiving pellets enriched with omega-3 FA. Hibernating mammals incorporate omega-6 FA into phospholipids of cellular membranes to survive lower body temperatures during hibernation and deep torpor [44]. The incorporation of omega-6-FA increases the activity of the sarcoplasmic reticulum calcium ATPase (SERCA), which is necessary to ensure proper calcium flux in cardiomyocytes at low tissue temperatures [45–47]. Similarly, it has been proposed that incorporation of omega-6 FA in peripheral skeletal muscle cells of red deer during winter ensures cell viability when extremities get very cold [13]. An increased omega-6 FA content of the feed also allowed for a more pronounced reduction in *f*_H_ during anaesthesia in this study.

The improvement of SERCA function could have increased cardiac contractility and thus, ABP in these animals [48]. The peripheral vasoconstriction associated with the decreased metabolic rate and body temperature could have contributed to this effect.

This study demonstrates that season, food restriction and dietary type of PUFA supplementation significantly influence *f*_H_ and ABP in female red deer anaesthetised with medetomidine-tiletamine-zolazepam.

Our results indicate, that seasonal physiological changes, as well as potentially altered drug pharmacokinetics and pharmacodynamics in highly seasonal mammals should be considered when anaesthetising such species. Improved differentiation between seasonal-, dietary, and drug-induced cardiovascular changes will help veterinarians to ensure the safest possible anaesthesia in red deer. Future studies, in both awake and anaesthetised animals, would be desirable to further disentangle the effects of seasonal acclimatisation and anaesthesia on the cardiovascular system of highly seasonal ungulates such as red deer.

## Supporting information

S1 Fig Seasonal differences of direct diastolic (A) and mean (B) arterial pressure of female red deer (*Cervus elaphus*). Deer (n = 11) were anaesthetised with 0.1 mg/kg medetomidine and 3 mg/kg tiletamine-zolazepam. Diastolic and mean arterial pressure were measured from minute 25 to 55 after anaesthesia induction (means ± standard error of the mean).
(PDF)

S2 Fig Seasonal differences during periods of unrestricted (*ad libitum*) and restricted food intake on direct diastolic (A) and mean (B) arterial pressure in female red deer (*Cervus elaphus*, n = 11). Diastolic and mean arterial pressure were measured from minute 25 to 55 after anaesthesia induction (means ± standard error of the mean). (See legend S1 Fig for medetomidine-tiletamine-zolazepam doses).
(PDF)

S3 Fig The influence of food regime (food restriction vs. *ad libitum*) and type of PUFA supplementation (omega-6 FA (n = 6) or omega-3 FA (n = 5) enriched pellets) on direct diastolic (A) and mean (B) arterial pressure in female red deer (*Cervus elaphus*, n = 11). Diastolic and mean arterial pressure were measured from minute 25 to 55 after initiation of anaesthesia (means ± standard error of the mean). (See legend S1 Fig for medetomidine-tiletamine-zolazepam doses).
(PDF)

S4 Fig The influence of altering type of PUFA supplementation (omega-6 FA (n = 6) or omega-3 FA (n = 5) enriched pellets) on direct diastolic (A) and mean arterial pressure in two different seasons (summer vs. winter) in female red deer (*Cervus elaphus*, n = 11). Diastolic and mean arterial pressure were measured from minute 25 to 55 after anaesthesia induction (means ± standard error of the mean). (See legend S1 Fig for medetomidine-tiletamine-zolazepam doses).
(PDF)

S1 Table Factors explaining the variation in direct mean arterial pressure of anaesthetised female red deer. Results of the best fitted linear mixed effect model explaining mean arterial pressure of female red deer (*Cervus elaphus*, n = 11) during anaesthesia with 0.1 mg/kg medetomidine and 3 mg/kg tiletamine-zolazepam. Animals were anaesthetised twice in winter (*ad libitum* and restricted feed) and twice in summer (*ad libitum* and restricted feed) and received pellets enriched with omega-6 FA (n = 6) or omega-3 FA (n = 5) enriched pellets.
(PDF)

S2 Table Factors explaining the variation in direct diastolic arterial pressure of anaesthetised female red deer. Results of the best fitted linear mixed effect model explaining diastolic arterial pressure of female red deer (*Cervus elaphus*, n = 11) during anaesthesia with 0.1 mg/kg medetomidine and 3 mg/kg tiletamine-zolazepam. Animals were anaesthetised twice in winter (*ad libitum* and restricted feed) and twice in summer (*ad libitum* and restricted feed) and received pellets enriched with omega-6 FA (n = 6) or omega-3 FA (n = 5) enriched pellets.
(PDF)
