# Supplementary material for: Effect of season and diet on heart rate and blood pressure in female red deer (Cervus elaphus) anaesthetised with medetomidine-tiletamine-zolazepam
Source: PLoS One. 2022 Jun 7;17(6):e0268811. doi: 10.1371/journal.pone.0268811 (PMC9173613; doi:10.1371/journal.pone.0268811)
Supplement: S1 Fig — Seasonal differences of direct diastolic (A) and mean (B) arterial pressure of female red deer (Cervus elaphus). Deer (n = 11) were anaesthetised with 0.1 mg/kg medetomidine and 3 mg/kg tiletamine-zolazepam. Diastolic and mean arterial pressure were measured from minute 25 to 55 after anaesthesia induction (means ± standard error of the mean). (PDF) [file pone.0268811.s001.pdf]

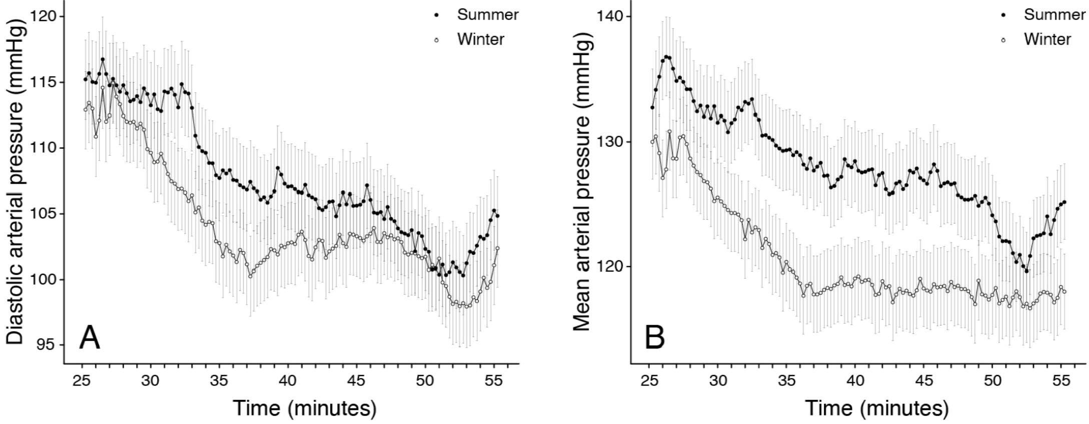

S1 Fig. Seasonal differences of direct diastolic (A) and mean (B) arterial pressure of female red deer (*Cervus elaphus*). Deer (n = 11) were anaesthetised with 0.1 mg/kg medetomidine and 3 mg/kg tiletamine-zolazepam. Diastolic and mean arterial pressure were measured from minute 25 to 55 after anaesthesia induction (means ± standard error of the mean).
