# Supplementary material for: Effect of season and diet on heart rate and blood pressure in female red deer (Cervus elaphus) anaesthetised with medetomidine-tiletamine-zolazepam
Source: PLoS One. 2022 Jun 7;17(6):e0268811. doi: 10.1371/journal.pone.0268811 (PMC9173613; doi:10.1371/journal.pone.0268811)
Supplement: S2 Fig — Seasonal differences during periods of unrestricted (ad libitum) and restricted food intake on direct diastolic (A) and mean (B) arterial pressure in female red deer (Cervus elaphus, n = 11). Diastolic and mean arterial pressure were measured from minute 25 to 55 after anaesthesia induction (means ± standard error of the mean). (See legend S1 Fig for medetomidine-tiletamine-zolazepam doses). (PDF) [file pone.0268811.s002.pdf]

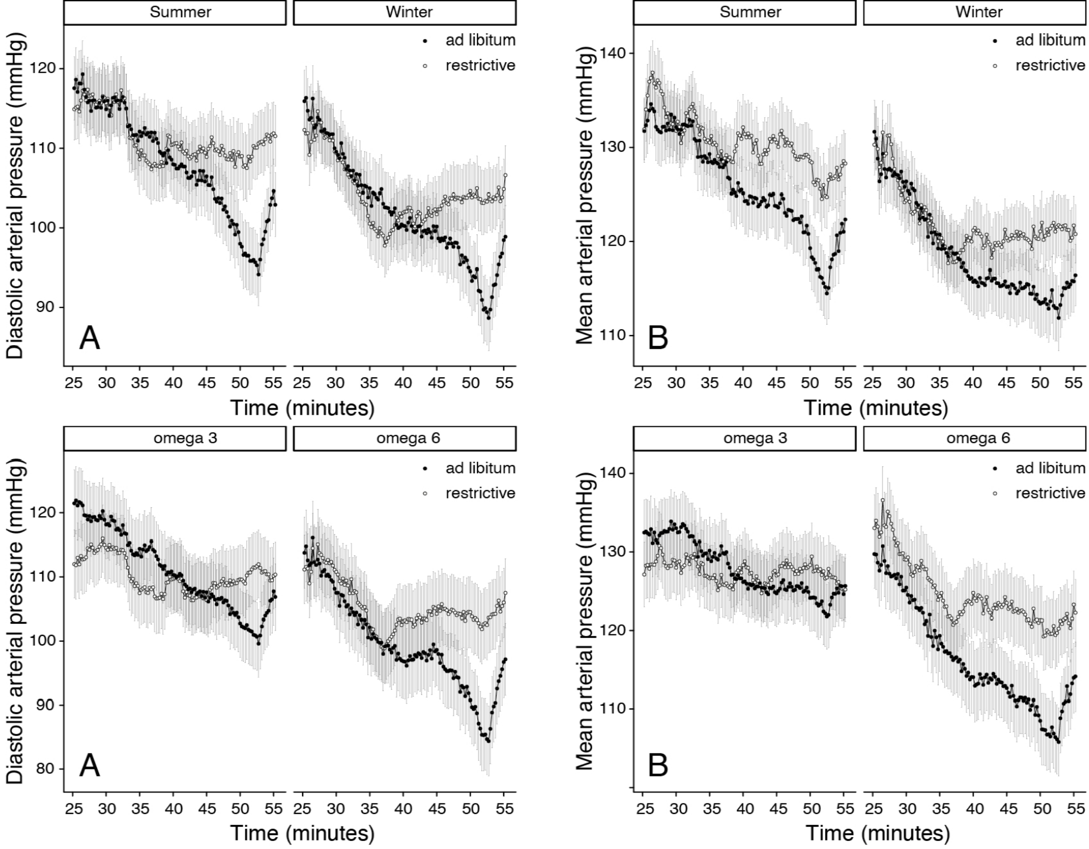

S2 Fig. Seasonal differences during periods of unrestricted (*ad libitum*) and restricted food intake on direct diastolic (A) and mean (B) arterial pressure in female red deer (*Cervus elaphus,* n = 11). Diastolic and mean arterial pressure were measured from minute 25 to 55 after anaesthesia induction (means ± standard error of the mean). (See legend S1 Fig for medetomidine-tiletamine-zolazepam doses).
