# Supplementary material for: Effect of season and diet on heart rate and blood pressure in female red deer (Cervus elaphus) anaesthetised with medetomidine-tiletamine-zolazepam
Source: PLoS One. 2022 Jun 7;17(6):e0268811. doi: 10.1371/journal.pone.0268811 (PMC9173613; doi:10.1371/journal.pone.0268811)
Supplement: S3 Fig — The influence of food regime (food restriction vs. ad libitum) and type of PUFA supplementation (omega-6 FA (n = 6) or omega-3 FA (n = 5) enriched pellets) on direct diastolic (A) and mean (B) arterial pressure in female red deer (Cervus elaphus, n = 11). Diastolic and mean arterial pressure were measured from minute 25 to 55 after initiation of anaesthesia (means ± standard error of the mean). (See legend S1 Fig for medetomidine-tiletamine-zolazepam doses). (PDF) [file pone.0268811.s003.pdf]

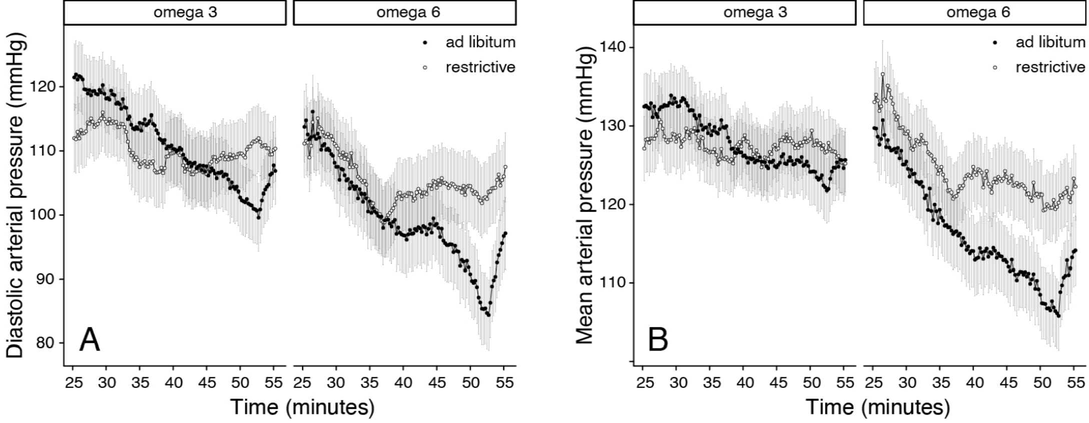

S3 Fig. The influence of food regime (food restriction vs. *ad libitum*) and PUFA composition (omega-6 FA (n = 6) or omega-3 FA (n = 5) enriched pellets) on direct diastolic (A) and mean (B) arterial pressure in female red deer (*Cervus elaphus,* n = 11). Diastolic and mean arterial pressure were measured from minute 25 to 55 after initiation of anaesthesia (means ± standard error of the mean). (See legend S1 Fig for medetomidine-tiletamine-zolazepam doses).
