# Supplementary material for: Effect of season and diet on heart rate and blood pressure in female red deer (Cervus elaphus) anaesthetised with medetomidine-tiletamine-zolazepam
Source: PLoS One. 2022 Jun 7;17(6):e0268811. doi: 10.1371/journal.pone.0268811 (PMC9173613; doi:10.1371/journal.pone.0268811)
Supplement: S1 Table — Results of the best fitted linear mixed effect model explaining mean arterial pressure of female red deer (Cervus elaphus, n = 11) during anaesthesia with 0.1 mg/kg medetomidine and 3 mg/kg tiletamine-zolazepam. Animals were anaesthetised twice in winter (ad libitum and restricted feed) and twice in summer (ad libitum and restricted feed) and received pellets enriched with omega-6 FA (n = 6) or omega-3 FA (n = 5) enriched pellets. (PDF) [file pone.0268811.s005.pdf]

**S1 Table. Factors explaining the variation in direct mean arterial pressure of anaesthetised female red deer.** Results of the best fitted linear mixed effect model explaining mean arterial pressure of female red deer (*Cervus elaphus,* n = 11) during anaesthesia with 0.1 mg/kg medetomidine and 3 mg/kg tiletamine-zolazepam. Animals were anaesthetised twice in winter (*ad libitum* and restricted feed) and twice in summer (*ad libitum* and restricted feed) and received pellets enriched with omega-6 FA (n = 6) or omega-3 FA (n = 5) enriched pellets.

| **Random effect** | **Std.Error** | | | |
|---|---|---|---|---|
| Subject | 9.19 | | | |
| **Predictor** | **Coefficient** | **Std.Error** | **Confidence Interval** | **p-value** |
| (Intercept) | 94.26 | 9.60 | 75.44 to 113.09 | < 0.001 |
| Food regime[restricted] | 1.02 | 1.14 | -1.21 to 3.25 | 0.371 |
| PUFA composition [omega-6 FA] | -13.93 | 5.93 | -27.35 to -0.51 | 0.043 |
| Season[Winter] | -6.85 | 1.48 | -9.74 to -3.96 | < 0.001 |
| Time | -0.09 | 0.01 | -0.1 to -0.08 | < 0.001 |
| Age | -0.96 | 1.07 | -3.07 to 1.14 | 0.368 |
| Body mass | 0.35 | 0.03 | 0.28 to 0.41 | < 0.001 |
| Food regime[restricted]*PUFA composition[omega-6 FA] | 9.02 | 1.21 | 6.65 to 11.39 | < 0.001 |
| Season[Winter]*Food regime[restricted] | -3.26 | 1.21 | -5.63 to -0.9 | 0.007 |
| Season[Winter]*PUFA composition[omega-6 FA] | 4.34 | 1.4 | 1.60 to 7.09 | 0.002 |

conditional $R^2$ = 88%, marginal $R^2$ = 44%
