# Supplementary material for: Effect of season and diet on heart rate and blood pressure in female red deer (Cervus elaphus) anaesthetised with medetomidine-tiletamine-zolazepam
Source: PLoS One. 2022 Jun 7;17(6):e0268811. doi: 10.1371/journal.pone.0268811 (PMC9173613; doi:10.1371/journal.pone.0268811)
Supplement: S2 Table — Results of the best fitted linear mixed effect model explaining diastolic arterial pressure of female red deer (Cervus elaphus, n = 11) during anaesthesia with 0.1 mg/kg medetomidine and 3 mg/kg tiletamine-zolazepam. Animals were anaesthetised twice in winter (ad libitum and restricted feed) and twice in summer (ad libitum and restricted feed) and received pellets enriched with omega-6 FA (n = 6) or omega-3 FA (n = 5) enriched pellets. (PDF) [file pone.0268811.s006.pdf]

S2 Table. Factors explaining the variation in direct diastolic arterial pressure of anaesthetised female red deer. Results of the best fitted linear mixed effect model explaining diastolic arterial pressure of female red deer (*Cervus elaphus,* n = 11) during anaesthesia with 0.1 mg/kg medetomidine and 3 mg/kg tiletamine-zolazepam. Animals were anaesthetised twice in winter (*ad libitum* and restricted feed) and twice in summer (*ad libitum* and restricted feed) and received pellets enriched with omega-6 FA (n = 6) or omega-3 FA (n = 5) enriched pellets.

Random effect	Std. Error			
Subject	6.13			
**Predictor**	**Coefficient**	**Std. Error**	**Confidence Interval**	**p-value**
(Intercept)	37.83	6.36	25.35 to 50.30	< 0.001
Food regime[restricted]	-8.5	0.87	-10.21 to -6.79	< 0.001
PUFA composition [omega-6 FA]	-7.54	3.97	-16.69 to 1.61	0.094
Season[Winter]	3.53	1.07	1.43 to 5.63	0.001
Time	0.02	0.01	0.00 to 0.03	0.021
Age	1.7	0.81	-0.17 50 3.56	0.069
Body mass	-0.05	0.01	-0.07 to -0.03	< 0.001
Food regime[restricted]*PUFA composition[omega-6 FA]	7.14	0.90	5.37 to 8.9	< 0.001
Season[Winter]*Food regime[restricted]	-2.85	0.97	-4.75 to -0.94	0.003
Season[Winter]*PUFA composition[omega-6 FA]	-2.59	0.92	-4.39 to -0.79	0.005
